# Towards Bioleaching of a Vanadium Containing Magnetite for Metal Recovery

**DOI:** 10.3389/fmicb.2021.693615

**Published:** 2021-06-30

**Authors:** Sören Bellenberg, Stephanie Turner, Laura Seidel, Nathan van Wyk, Ruichi Zhang, Varvara Sachpazidou, Rodrigo F. Embile, Ingar Walder, Tiina Leiviskä, Mark Dopson

**Affiliations:** ^1^Centre for Ecology and Evolution in Microbial Model Systems (EEMiS), Linnaeus University, Kalmar, Sweden; ^2^Chemical Process Engineering, University of Oulu, Oulu, Finland; ^3^Kjeøy Research & Education Center, Vestbygd, Norway

**Keywords:** vanadium, magnetite, *Gluconobacter oxydans*, 16S rRNA amplicon sequencing, bioleaching

## Abstract

Vanadium – a transition metal – is found in the ferrous-ferric mineral, magnetite. Vanadium has many industrial applications, such as in the production of high-strength low-alloy steels, and its increasing global industrial consumption requires new primary sources. Bioleaching is a biotechnological process for microbially catalyzed dissolution of minerals and wastes for metal recovery such as biogenic organic acid dissolution of bauxite residues. In this study, 16S rRNA gene amplicon sequencing was used to identify microorganisms in Nordic mining environments influenced by vanadium containing sources. These data identified gene sequences that aligned to the *Gluconobacter* genus that produce gluconic acid. Several strategies for magnetite dissolution were tested including oxidative and reductive bioleaching by acidophilic microbes along with dissimilatory reduction by *Shewanella* spp. that did not yield significant metal release. In addition, abiotic dissolution of the magnetite was tested with gluconic and oxalic acids, and yielded 3.99 and 81.31% iron release as a proxy for vanadium release, respectively. As a proof of principle, leaching via gluconic acid production by *Gluconobacter oxydans* resulted in a maximum yield of 9.8% of the available iron and 3.3% of the vanadium. Addition of an increased concentration of glucose as electron donor for gluconic acid production alone, or in combination with calcium carbonate to buffer the pH, increased the rate of iron dissolution and final vanadium recoveries. These data suggest a strategy of biogenic organic acid mediated vanadium recovery from magnetite and point the way to testing additional microbial species to optimize the recovery.

## Introduction

Vanadium is a transition metal that is primarily used as a steel alloy in approximately 85% of global steel production. It is also used in the aerospace industry for the production of alloyed-titanium, in industrial catalysts for the production of synthetic products, and in the cathodes of some lithium ion batteries [reviewed in Peng (2019); Gilligan and Nikoloski (2020)]. Vanadium is widespread in the Earth’s crust but it does not form concentrated deposits, such as a sulfide mineral, but occurs rather as V(III) replacing iron or aluminum in different minerals including titanomagnetite (Dill, 2010). The majority of vanadium is refined from slag waste as a by-product of the processing of titanomagnetite ores and steel refining, and to a lesser extent as a by-product of uranium mining [reviewed in Gilligan and Nikoloski (2020)]. In addition, vanadium is found in coal, oil shale, phosphate rock, and crude oil. A general process for vanadium production can include magnetic separation, salt roast, water leach, precipitation, and calcination of V_2_O_5_. Up to 87% vanadium recovery from magnetite is achieved by chemical leaching using a mixture of nitric and sulfuric acids at a temperature of 80–95°C (Nejad et al., 2018). A further study used a combined process involving magnetic separation, hydrofluoric acid leaching, co-precipitation, roasting, water leaching, and precipitation to achieve a total vanadium recovery of 81% (purity of >99%) from titanomagnetite (Zhu et al., 2016). The use of vanadium in next-generation energy storage and conversion technologies is predicted to increase its demand and highlights the need to develop production methods (Zhang et al., 2020).

Due to increased extraction and use along with its significant environmental toxicity, vanadium’s position as an environmental hazard is being reconsidered (Watt et al., 2018). However, knowledge of vanadium geochemistry is lacking in comparison to other environmental pollutants. Vanadium exists in the +3, +4, and +5 oxidation states and is most common as the +5 vanadate oxyanion in oxic conditions, while the +3 state occurs in anoxic/sulfidic conditions (Gustafsson, 2019; Shaheen et al., 2019). Vanadium transfers from soil to plants such that phytoaccumulation may impact human health by potentially causing a number of diseases (Imtiaz et al., 2015). A study in China identified vanadium in soils and groundwater where species from the *Bacillus* and *Thauera* genera were well represented, resulting in reduction of V^5+^ to less toxic V^4+^ suggesting a path to bioremediation with these species (Zhang et al., 2019). Vanadium can also be recovered by chemical and biological sorbents for both remediation and industrial vanadium recovery (Huang et al., 2020; Kong et al., 2020; Zhang and Leiviskä, 2020; Sharififard and Rezvanpanah, 2021). However, the paucity of microbiological studies highlights the gap in current knowledge of vanadium-contaminated environments.

Biomining is the use of microbes to catalyze the dissolution of solid metal-containing minerals into metal-containing solutions [reviewed in Johnson (2014)]. This metal dissolution is often facilitated by low pH in combination with microbial catalyzed oxidation (Vera et al., 2013) or reduction (Johnson and Du Plessis, 2015) of iron ions. Several metal sulfide ore biomining systems have been developed with the most well-known being the low pH microbial catalyzed oxidative dissolution of metal sulfides, such as copper release from chalcopyrite (Watling, 2006; Christel et al., 2018). More recent technologies have been developed including the reductive dissolution of e.g., limonitic laterite by acidophilic microorganisms for the recovery of nickel (Hallberg et al., 2011). *Shewanella* spp. are known to use magnetite as an electron acceptor (Kostka and Nealson, 1995) and the reductive bioleaching of several iron(III) oxides including magnetite by *Shewanella loihica* shows dissimilatory iron reduction and dissolution of all the tested minerals (Benaiges-Fernandez et al., 2019). The solubility of metals can also be enhanced by heterotrophic microbes that oxidize organic substrates (such as glucose to organic acids) that are excreted and act as metal-binding ligands (Pohlman and Mc Coll, 1986; Jones et al., 1996). Consequently, biological extraction of metals using organic acid producing fungi and bacteria (e.g., *Gluconobacter* spp. producing gluconic acid) has also been investigated (Bosecker, 1997; Mulligan et al., 2004). These studies include base metal recycling from electronic waste by e.g., fungal ligands (Valix, 2017) and rare earth element release from e.g., bauxite residues by bacterial and fungal biogenic organic acids [reviewed in Rasoulnia et al. (2020)]. Bioleaching strategies have also been tested for vanadium recovery including oxidative and organic acid leaching from spent catalysts by *Acidithiobacillus ferrooxidans*, *Acidithiobacillus thiooxidans*, and *Aspergillus niger* (Muddanna and Baral, 2019; Mikoda et al., 2020; Pradhan et al., 2021); shale by *At. ferrooxidans* (He et al., 2019); red mud with the fungi *A. niger* and *Penicillium tricolor* (Qu et al., 2019); and steel slag by a mixed consortium of acidophilic bacteria dominated by *At. ferrooxidans* and *At. thiooxidans* (Gomes et al., 2018). However, the paucity of knowledge regarding the biological recovery of vanadium from the industrially relevant resource of titanomagnetite highlights the need to develop biotechnologies for a more sustainable recovery of vanadium from magnetite-bearing sources.

In this study, we investigated the 16S rRNA gene amplicon-based molecular microbiology of environments with industrially relevant vanadium concentrations to inform strategies in the development of a biomining technology. To this end, several chemical and biological mineral dissolution systems were tested to identify the most efficient method to release vanadium from magnetite concentrate for metal recovery.

## Materials and Methods

### Characterization of Solid and Liquid Samples

Waste rocks samples (BRU_S1 to S6; *n* = 6) were collected from the Bruvann mine by combining material from the upper 15 cm of the waste dump that were used for mineralogical analyses (Supplementary File 1). Magnetite was extracted from the rocks by magnetic separation to run the laboratory leaching test on the material for the microbial analysis of sample BRU_L4 (described below in section “Microbial Sample Collection, DNA Extraction, and 16S rRNA Amplicon Analysis”). In addition, solid waste (MV_S1 and S2; *n* = 2) was collected from the upper 15 cm of the Mustavaara tailings site. Solid samples were ground and pressed into pellets and then analyzed by X-Ray Fluorescence Spectroscopy (XRF) using a Bruker AXS S4 Pioneer machine. The MV_L1 and L2 liquid samples were analyzed with an Analytik Jena Contra 700 atomic absorption spectrometer with a graphite furnace while the Ti_L1 liquid sample was analyzed for vanadium concentration by inductively coupled plasma mass spectrometry (ICP-MS) using a Thermo Fisher Scientific iCAP RQ machine according to ISO 17294-2:2016. Samples BRU_L1 to L3 pH and conductivity were measured in the field using an Aqua TROLL multi parameter sonde while BRU_L4 was measured using an OAKTON pH 2100 and a HACH sensION 5, respectively. Conductivity and pH of Ti_L1 plus MV_L1 and L2 liquid samples were analyzed using a Mettler Toledo conductivity meter and a pHenomenal^®^ pH 1000 L (VWR) pH meter, respectively.

### Microbial Sample Collection, DNA Extraction, and 16S rRNA Amplicon Analysis

Water, sediment, and solid waste samples were collected for microbial community analyses at vanadium-containing sites in Norway and Finland in 2016 and 2018 with sample details provided in Supplementary File 1. Bruvann mine sediment samples were collected from a mine stream (BRU_S7 and S8; *n* = 2) by combining material from the upper 15 cm below the sediment surface. In addition, Mustavaara solid tailings waste (MV_S3 to S8; *n* = 6) was sampled from the tailing area (next to the settling basin) by first removing approximately 20 cm of the top layer of the tailing material and then taking all samples from approximately same depth. Water samples were obtained from the Bruvann mine stream as close to the mine entrance as possible (samples BRU_L1 and L2; *n* = 2), Bruvann tailings deposit stream leading from the mine tailings deposit (BRU_L3; *n* = 1), Bruvann leaching experiment (BRU_L4; *n* = 1), Mustavaara settling basin (MV_L3 to L5; *n* = 3), and Titania mine water (Ti_L1; *n* = 1). All the water samples were separately filtered (0.1 μm) for cell recovery and each filter was placed in a sterile tube.

The filters, sediment, and solid waste samples were frozen and stored at −20 or −80°C until analysis. Genomic DNA was extracted using the PowerWater DNA isolation Kit (Qiagen) for filters and PowerSoil DNA isolation Kit (Qiagen) for sediment and solid tailings waste samples according to the manufacturers’ instructions other than that the DNA was eluted in 50 μL of EB buffer.

Partial 16S rRNA genes were amplified with a modified PCR protocol (Hugerth et al., 2014) by using the 341F and 805R primer set (Herlemann et al., 2011). The PCR amplification and Illumina libraries were constructed and sequenced by Science for Life Laboratory, Sweden^1^ according to published methods (Lindh et al., 2015). The DADA2 pipeline [version 1.16, “dada2” R package version 1.14.1; (Callahan et al., 2016)] was used to process the sequence data. After initial filtering and trimming (280 and 220 bp sequence length for forward and reverse reads, respectively; 21 bp trimmed for primers), the remaining primer sequences were removed with cutadapt [version 2.3; (Martin, 2011)]. On average, 18% of the raw reads were filtered out. The resultant amplicon sequence variants (ASVs) were annotated against the Silva NR database v138 (Quast et al., 2013), and analyzed in R [version 3.6.3; (R Core Team, 2019)] utilizing the vegan package [version 2.5–6; (Oksanen et al., 2020)].

### Magnetite Concentrate

Magnetite concentrate was provided by Titania AS (Norway). A Bruker AXS S4 Pioneer X-ray fluorescence (XRF) spectrometer was used to determine the chemical composition of the magnetite concentrate. The sample was milled and 13.16 g was mixed with 0.84 g of C-wax and pressed pellets were prepared from the mixture (7–8 g) using boric acid as binder and applying a hydraulic pressure of 10 metric tons to pellet the sample. X-ray diffraction (XRD) measurement was performed with a Rigaku SmartLab rotating anode diffractometer using Co Ka radiation. The milled sample was measured at room temperature in the 2-theta range from 5° to 130° with a step size of 0.02 and an acquisition rate of 4 deg/min. Rietveld analysis was performed to measure the proportion of crystalline compounds. X-ray photoelectron spectroscopy (XPS) analyses were performed with a Thermo Fisher Scientific ESCALAB 250Xi using a monochromatic Al Kα source (1486.6 eV). The charge correction was done by setting the binding energy (BE) of adventitious carbon to 284.8 eV.

### Abiotic Organic Acid Leaching

Abiotic leaching using the metal-binding ligands gluconic acid (0.5 N, 500 mM) and oxalic acid (0.5 N, 250 mM) was performed in triplicate assays using 100 mL at pH 1.8 in 250 mL Erlenmeyer flasks with agitation at 120 rpm and 30°C. Samples of the leach were removed and analyzed as described below [see section “(Bio)leaching Analyses”].

### Bioleaching Experiments With Iron(III)-Reducing Acidophiles

Reductive bioleaching with iron(III)-reducing acidophiles used mixed cultures of the iron- and sulfur-oxidizing species *At. ferrooxidans* ATCC 53993, the sulfur-oxidizer *At. thiooxidans* DSM 9463, and the heterotroph *Acidiphilium cryptum* JF-5 [kindly provided by Küsel et al. (1999, 2002)]. *At. ferrooxidans* and *At. thiooxidans* strains were grown axenically in 250 mL Erlenmeyer flasks with 100 mL Mackintosh (Mac) basal salt medium (Mackintosh, 1978) at pH 2.5 with 1% (wt/vol) elemental sulfur. *Ac. cryptum* JF-5 was grown in 100 mL Erlenmeyer flasks with 50 mL Mac medium supplemented with 1 g/L glucose and 0.02% (wt/vol) yeast extract. The cultures were grown at 30°C with agitation (120 rpm). Early stationary phase cultures were used as inocula for bioreactor experiments performed in 2 L Schott flasks with 2 L medium supplemented with 0.5% elemental sulfur and 1 g/L of iron, supplied as Fe_2_SO_4_ × 7H_2_O solution (pH 1.2) and stirred at 300 rpm using a magnetic stirrer. The bioreactors were initially operated with aeration until the pH dropped below 1.2 before aeration was stopped, the pH was set to 2.0 with 2 M NaOH, the medium was purged with nitrogen and supplemented with 3% (wt/vol) Titania magnetite concentrate and ingress of oxygen was prevented by rubber seals. The aerobic metabolism of the microorganisms was initially used for efficient growth and then for oxygen elimination in order to switch from oxygen to iron respiration. Sampling was via sterile needles during which nitrogen was injected into the reactors to prevent oxygen ingress. Bioleaching parameters were sampled and analyzed as described below [see section “(Bio)leaching Analyses”].

### Magnetite Reduction With *Shewanella loihica*

Strain PV-4 (DSM 17748) was pre-cultured aerobically in 100 mL Erlenmeyer flasks with 50 mL LB medium at 20°C with agitation (120 rpm). Cells were harvested by centrifugation at 7,000 × *g* for 10 min and washed in M1 defined medium (Roh et al., 2006). The reduction of magnetite was assessed in 100 mL serum bottles using 100 mL M1 medium supplemented with lactate (10 mM) as the electron donor and 3% (wt/vol) Titania magnetite concentrate. Alternatively, the reduction of magnetite was tested in synthetic seawater prepared following the standard protocol D1141-98 (ATSM International) amended with sodium lactate (10 mM) as an electron donor and carbon source, ammonium chloride (1.87 mM) as a source of nitrogen, and Tris–HCl (10 mM) as a pH buffer (Benaiges-Fernandez et al., 2019). The pH of the medium was adjusted to 8.2 with 0.1 N NaOH. Reduction of magnetite was conducted anaerobically in N_2_ purged medium in sealed serum bottles. Bioleaching cultures were sampled and analyzed as described below [see section “(Bio)leaching Analyses”].

### Magnetite Bioleaching With *Gluconobacter oxydans*

*Gluconobacter oxydans* strain DSM 46616 was pre-grown in DSM 105 medium (glucose 100 g/L, yeast extract 10 g/L, CaCO_3_ 20 g/L, pH 6.8) at 30°C and 120 rpm agitation. For magnetite leaching assays, sub-cultures were supplemented with 3% (wt/vol) Titania magnetite concentrate and inoculated with 10% (vol/vol) pre-grown culture. Different medium compositions were tested by modifying DSM 105 medium with lower yeast extract concentration (0.1% compared to 1%) or omitting/adding CaCO_3_ as described for the different experiments. Lowering the yeast extract content was performed to investigate if lowered levels of yeast extract (i.e., growth factors and nitrogen) in the medium fostered conversion of glucose to gluconic acid, since conversion into biomass would be limited. Bioleaching cultures were sampled and analyzed as described in section “(Bio)leaching Analyses.”

### (Bio)leaching Analyses

The parameters tested in the chemical and biological leaching were pH (VWR^TM^ pHenomenal pH1000L, SI Analytics BlueLine 15pH probe), redox potential (VWR pHenomenal^TM^ pH1100L, Mettler Toledo InLab Redox-L), as well as photometric determination of iron(II)-ions and total iron ion concentrations (Harvey et al., 1955) using a plate reader (FLUOstar Omega^TM^, BMG LabTech^®^). Cell counts were performed with a Thoma counting chamber on a Zeiss microscope with a magnification of 400-fold. Selected samples were analyzed for their total vanadium content (ICP-MS) as carried out at ALS AB, Sweden or Servizos de Apoio á Investigación, Spain.

## Results and Discussion

### Characterization of Vanadium Containing Environments

Nordic vanadium containing environments including operating and closed mines were sampled to investigate indigenous microbial communities that may provide information into leaching strategies for vanadium dissolution. The closed Bruvann nickel-olivine deposit has six Mtons of deposited tailings and while no vanadium data are available for the near pH neutral liquid samples used for microbial community analysis, Bruvann waste rocks (BRU_S1 to BRU_S6) contained vanadium concentrations ranging from 59 to 161 ppm along with 35–45% (wt/wt) SiO_2_ indicating the samples were dominated by felsic minerals and high in olivine (Supplementary File 2). The Mustavaara Fe-Ti-V-rich magnetite deposit mine has resulted in vanadium contaminated surrounding natural water systems (ponds, lakes, and rivers) along with vanadium containing tailings. The settling basin MV_L1 and L2 water samples contained between 9.42 and 9.74 μg/L vanadium while the vanadium content in the tailings (MV_S1 and S2) was 1,029 and 755 ppm, respectively. Finally, the Ti_L1 Titania mine water contained 526 μg/L of vanadium, high sulfate, and low pH that was loaded with many constituents in addition to vanadium including 219 mg/L iron enriched due to sulfide oxidation in the drying process of Ni-Cu sulfide concentrate.

### Microbial Diversity of Vanadium Containing Environments

The rarefaction analysis generally showed asymptote converged curves that indicated the majority of taxa were captured by the sequencing (Supplementary File 3). However, samples MV_L5 and MV_S5 did not fully reach the horizontal curve progression, likely due to the low number of obtained sequences for these two samples. The similar values for observed ASVs (“Observed”) and estimated richness (“Chao1”) confirmed a sufficient sequencing depth for most samples (Supplementary File 4). The average number of observed ASVs per sample was 1946 ± 2047 (min 185 and max 5972). The greatest Alpha diversity as determined by Chao1 richness and Shannon diversity index was observed in Bruvann (BRU_L3 and BRU_L1) and Mustavaara (MV_L4) mine water samples with values ranging from 4,854 to 5,977 (Chao1) and 6.87 to 7.85 (Shannon). The increased diversity of the water samples may have been due to the low number of niches and relative scarcity of electron donors present in the tailings samples.

**FIGURE 1:**
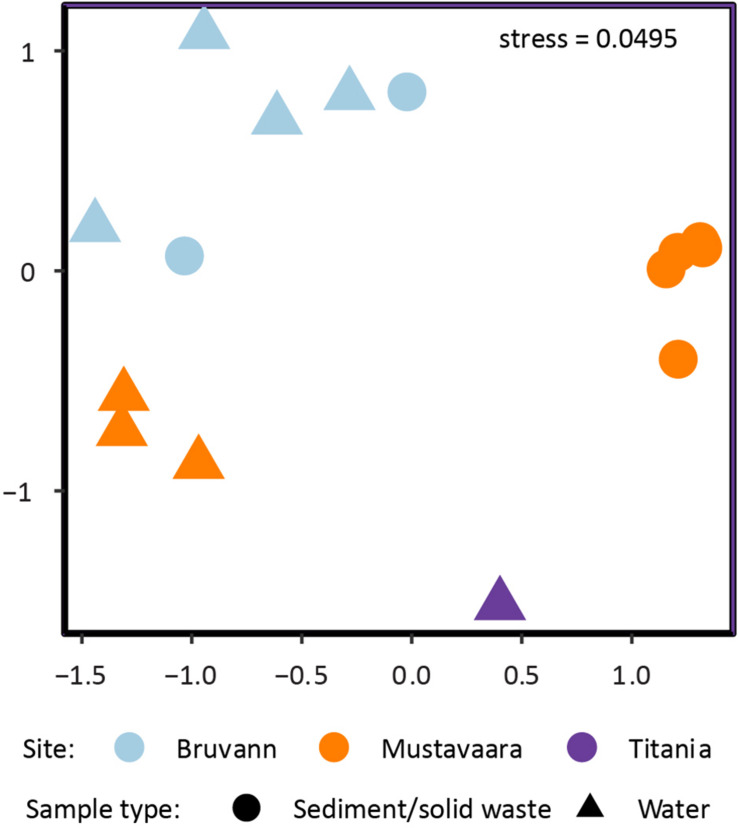
Non-metric multidimensional scaling (NMDS) of microbial community composition for the environmental 16S rRNA gene samples. The NMDS is based on a Bray Curtis dissimilarity matrix using relative abundances of the ASVs. Color encodes sampling site and symbols encode sample type.

Non-metric multidimensional scaling (NMDS) comparison of microbial community composition between samples (Beta diversity; Figure 1) showed similar communities within groups for e.g., Mustavaara solid waste samples while the respective mine water samples were dissimilar. Differences in the Mustavaara communities may be related to low interface area between fluid and solid waste samples, different sampling areas, or the different sampling times (i.e., water samples were taken in October 2017, whereas solid waste samples were taken in August 2018). The Titania microbial communities (Ti_L1) were most dissimilar to those of the other samples potentially as the site is located in the south of Norway compared to the northern sites for the other samples plus the Titania mine water was acidic compared to near pH neutral conditions in the other samples.

### Taxonomic Analysis of the Vanadium Containing Communities

**FIGURE 2:**
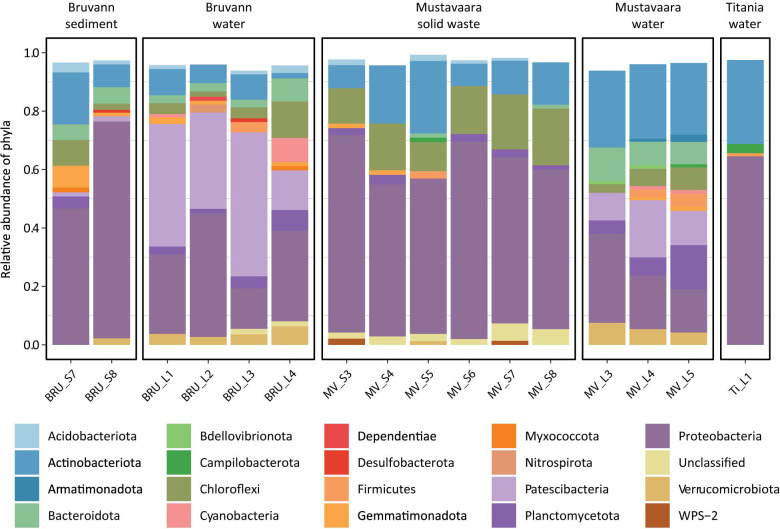
Stacked bar graph of the microbial community composition based on the relative abundances of phyla (>1%). The remaining proportion to 100% (1.0) includes low-abundant taxa of <1% of the relative abundance.

Overall, the most abundant 16S rRNA gene amplicons had sequences that aligned within the phyla Proteobacteria, Patescibacteria, Actinobacteriota, and Chloroflexi (Figure 2 with taxonomic levels to genera in Supplementary File 5).

Patescibacteria and Proteobacteria dominated the oxic Bruvann mine water samples (BRU_L1 to BRU_L4). The Proteobacteria included *Thiobacillus* and *Sulfurifustis* that include autotrophic sulfur-oxidizers (Vishniac, 1952; Kojima et al., 2015), the iron-oxidizing *Sideroxydans* (Weiss et al., 2007), and the genus *Rhodoferax* that includes psychrophilic aerobes that can grow on glycerol, mannose, and mannitol (Kaden et al., 2014). In contrast, the class Gammaproteobacteria dominated the likely anoxic Bruvann BRU_S7 and BRU_S8 sediment samples that included sulfur-oxidizing and nitrate-reducing *Thiobacillus* and *Sulfuriferula* (Watanabe et al., 2015), the psychrophilic and facultative anaerobic *Rhodoferax*, and *Actimicrobium* that was isolated from Antarctic seawater (Kim et al., 2011). The identification of ASVs most similar to psychrophilic bacteria matches the low temperatures typically encountered in northern Norway and the facultative aerobes match the likely oxygen concentrations in the sediment samples.

The Mustavaara mine tailings water samples (MV_L3 to MV_L5) were dominated by the phyla Actinobacteriota, Proteobacteria, Patescibacteria, and Bacteroidota with the respective classes Actinobacteria, Gammaproteobacteria, Parcubacteria, and Bacteroidia. Proteobacteria dominated the Mustavaara solid waste samples (MV_S3 to MV_S8) with lower relative abundances of Chloroflexi and Actinobacteriota. Within the class Gammaproteobacteria, the genus *Gallionella* was abundant that includes iron-oxidizing species that reside in low oxygen environments (Hallbeck and Pedersen, 2015).

The Titania mine water (Ti_L1) microbial community was similar to the Mustavaara solid waste samples on phylum and class levels but was different on more defined taxonomic levels. The most abundant genera within the Gammaproteobacteria were *Pseudomonas* (family Pseudomonadaceae), *Polynucleobacter* (family Burkholderiaceae), as well as *Hydrogenophaga*, and *Limnohabitans* (family Comamonadaceae). In addition, this water sample contained 16S rRNA gene sequences that aligned within the *Gluconacetobacter* genus that typically produces ketogluconic acid (Yamada et al., 1997) and the acidophilic *Acidithiobacillus* genus that can mediate sulfur compound oxidation along with some species also oxidizing iron (Johnson, 2016).

These vanadium-containing magnetite environments contained populations in the water samples and to some extent the sediment/tailings samples typical for pH neutral (neutrophiles), low temperature (psychrophiles and psychrotolerant taxa), and oxic versus anoxic environments. In addition, populations that have the potential to be used for mineral dissolution were present such as the acidophilic *Acidithiobacillus* genus that may be able to mediate low pH reductive bioleaching (Hallberg et al., 2011; Smith et al., 2017) or the *Gluconacetobacter* genus for organic acid dissolution [reviewed in Rasoulnia et al. (2020)]. The presence of these microbes in the oxic and anoxic environments that have previously been demonstrated to mediate metal dissolution led to testing of several strategies for vanadium dissolution.

### Magnetite Concentrate Composition

The chemical composition determined by XRF confirmed iron as the main element along with significant amounts of titanium, magnesium, aluminum, silicon, and chromium plus a vanadium content of 3739 ppm (Supplementary File 6). XRD analysis showed that the magnetite concentrate contained mostly magnetite (∼90%; Mg_0.04_Fe_2.96_O_4_) and a small amount of magnesium titanium oxide (∼5%; MgTiO_3_; Supplementary File 7). The third phase present in the sample was not accurately identified, but data analysis indicated a spinel phase of spinel ferrian chromian or chlorospinel. The XPS survey spectrum confirmed the presence of O, Fe, Si, Mg, S, C, Al, Na, and Ti on the surface of the magnetite sample (Supplementary File 8). Vanadium was not detected in the survey and neither in the V2p high-resolution spectrum, which indicated that vanadium was buried deeper in the sample. The Fe2p high-resolution spectrum showed the Fe2p_3/2_ peak at 711.8 eV and the Fe2p_1/2_ peak at around 725 eV (Supplementary File 9). The binding energies are consistent with previous studies related to magnetite characterization with XPS (Tian et al., 2011; Cuenca et al., 2016). No clear satellite peak was observed at around 719 eV, which further confirmed that the sample contained magnetite (Cuenca et al., 2016). Ti2p spectrum showed doublet peaks of Ti2p_3/2_ at 459.0 eV and Ti2p_1/2_ at 464.8 eV (Supplementary File 9) indicating that Ti existed in +4 state (Pukazhselvan et al., 2019) that is also the oxidation state of Ti in MgTiO_3_.

### Acidophile Bioleaching of the Vanadium Containing Magnetite Concentrate

Iron(III) reducing acidophiles, namely *At. ferrooxidans*, *At. thiooxidans* (Marrero et al., 2015, 2017), and *Ac. cryptum* JF-5 (Harvey et al., 1955) were investigated for magnetite bioleaching tests. These species are able to reduce iron(III) and are consequently capable of reductive bioleaching (Küsel et al., 1999, 2002; Hallberg et al., 2011; González et al., 2015; Marrero et al., 2017). Bioreactors (2 L) inoculated with the three species achieved low magnetite dissolution (1.74 ± 0.12%; Table 1) within 45 days of cultivation as well as shake flask experiments that were incubated aerobically or anaerobically. Likewise, axenic shake flask experiments with *Ac. cryptum* that were also conducted under aerobic or anaerobic conditions yielded low magnetite dissolution (0.39 ± 0.09 or 0.45 ± 0.05%; Table 1) and were therefore deemed unsuitable for vanadium leaching from the tested mineral concentrate. Other studies have reported more efficient magnetite dissolution by *A. cryptum* JF-5, reaching up to 25% in anaerobic and pH-controlled systems (González et al., 2015) although these conditions are more difficult and expensive to be implemented in large scale industrial biohydrometallurgical processes.

**TABLE 1:** Summary table of magnetite dissolution from the (bio)leaching strategies tested.

	Iron yield		Rate	Leaching time	Magnetite dissolution
		
	mmol/L	mg/L	mmol L^–1^ d^–1^	Days	%
**Chemical leaching (this study)**					
Gluconic acid (0.5 N)	14.0	783	0.93	15	3.99 ± 0.03
Oxalic acid (0.5 N)	285.3	15 930	47.5	6	81.31 ± 1.71
**Bioleaching (this study)**					
Acidophile bioreactor (anaerobic)	6.1	341	0.14	45	1.74 ± 0.12
*A. cryptum* (aerobic)	1.4	77	0.03	40	0.39 ± 0.09
*A. cryptum* (anaerobic)	1.6	88	0.04	40	0.45 ± 0.05
*G. oxydans* (aerobic)	32.2	1 799	2.15	16	9.18 ± 0.16
**Bioleaching (previous studies)**					
*S. loihica* [anaerobic medium (Roh et al., 2006)]	2.0	113	0.04	46	0.58 ± 0.03
*S. loihica* [anaerobic medium (Benaiges-Fernandez et al., 2019)]	0.5	28	0.01	87	0.14 ± 0.04

### *Shewanella loihica* Bioleaching of the Magnetite Concentrate

As mentioned above, *Shewanella* spp. use magnetite as an electron acceptor during dissimilatory microbial reduction (Kostka and Nealson, 1995) and magnetite reduction has recently been demonstrated to occur under marine sediment conditions (Benaiges-Fernandez et al., 2019). Consequently, bioleaching by *S. loihica* PV-4 was tested for its potential to dissolve the magnetite concentrate. The yields of iron obtained in this study using marine and non-marine media plus lactate as electron donor (0.14 ± 0.04 and 0.58 ± 0.03%, respectively) were greater than the 0.05% magnetite dissolution obtained by *S. loihica* under marine conditions (Benaiges-Fernandez et al., 2019). Despite these slight increases in iron concentration (Table 1), the low rates and yields render this option unfeasible for the development of an industrial bioleaching process.

### Abiotic Organic Acid Leaching of the Magnetite Concentrate

**FIGURE 3:**
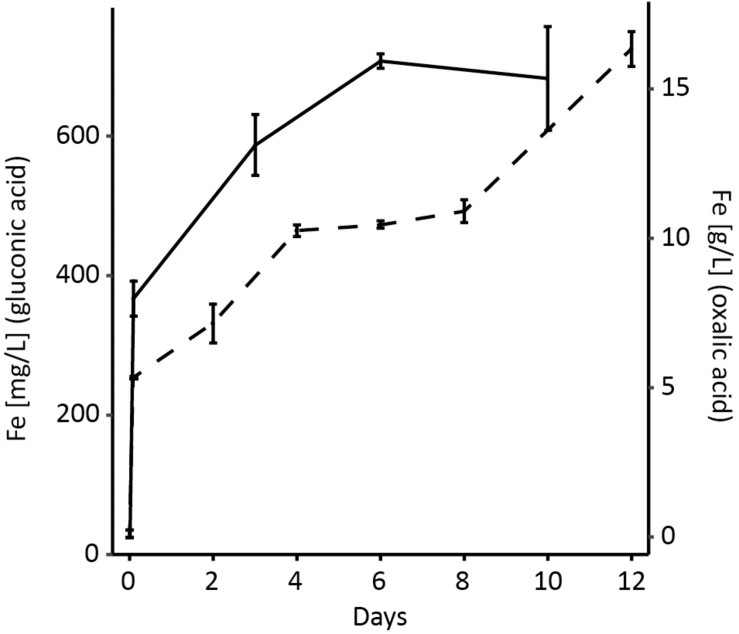
Chemical leaching of magnetite using gluconic acid (dashed line) and oxalic acid (solid line). Equal normality (0.5 N) solutions of gluconic and oxalic acid were used in shake flasks incubated at 30°C and 120 rpm agitation. Data are averages ± SD (*n* = 3).

Chemical leaching of the magnetite concentrate was tested with organic acids (0.5 N, pH 1.8) to investigate their efficacy for magnetite dissolution [reviewed in Eisele and Gabby (2014)]. Iron leached from the magnetite reached 14.0 and 285.3 mmol/L during gluconic- and oxalic-acid leaching after 15 and 6 days, respectively (Figure 3 and Table 1). This corresponded to 3.99 ± 0.03 and 81.31 ± 1.71% of the available iron in the 3% (wt/vol) magnetite pulp density in the shake flasks. Consequently, the dibasic oxalic acid was a more potent ligand than the monobasic gluconic acid and therefore, was confirmed to be suitable for magnetite dissolution (Lee et al., 2007). Both organic acids gave significantly higher yields (Table 1) than the acidophile and *S. loihica* bioleaching described above. Therefore, investigations into whether biological organic acid production also increased the magnetite dissolution were carried out. *A. niger* has been successfully applied for iron removal from silicates (Castro et al., 2000) and clay (Mandal and Banerjee, 2004) via binding by e.g., citric, oxalic, malic, and gluconic acids. In addition, it has been used to recover valuable metals from a low-grade mining ore (Mulligan et al., 2004); bioleaching of metals from spent fluid catalytic cracking catalyst (Santhiya and Ting, 2006; Das et al., 2019), waste printed circuit boards (Faraji et al., 2018), and spent lithium ion batteries (Horeh et al., 2016). Many of these studies reported that biogenic organic acids (culture supernatants) outperformed chemical leaching of comparable assays with pure organic acids (Reed et al., 2016; Qu et al., 2019).

### *Gluconobacter oxydans* Bioleaching of the Magnetite Concentrate

**FIGURE 4:**
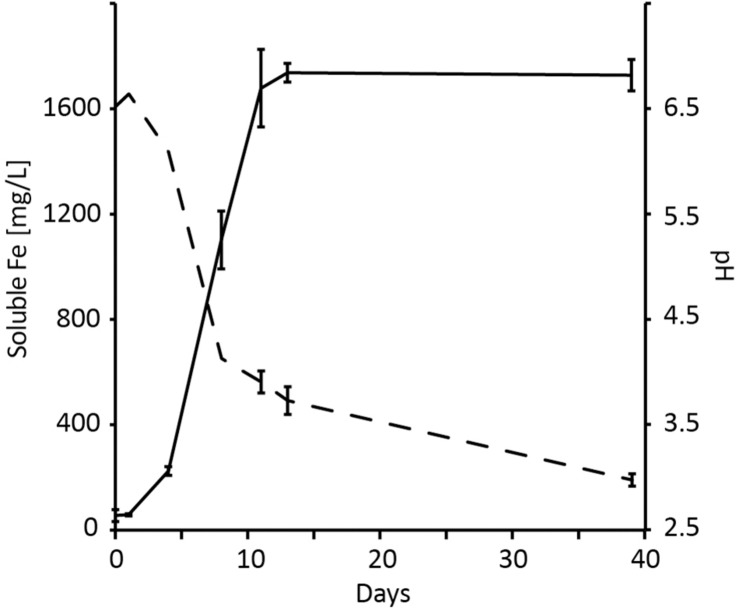
Bioleaching of 3% (wt/vol) magnetite concentrate using *Gluconobacter oxydans* showing the development of soluble iron ions (solid line) and pH (dashed line). Data are averages ± SD (*n* = 3) except for pH values between days 0 and 4 that are single replicates.

*Gluconobacter* spp. produce the ligand gluconic acid when grown with glucose as substrate (Gupta et al., 2001). Consequently, biogenic gluconic acid was used to test metal solubilization from solids as a proof of concept for vanadium recovery from Titania magnetite concentrate. An initial test of *G. oxydans* bioleaching efficacy in assays with unamended medium containing 0.1% (wt/vol) yeast extract yielded 1737 ± 36 mg/L (*n* = 3) soluble iron after 13 days that corresponded to 8.9% of the available iron in the magnetite concentrate (Figure 4). During bioleaching, the pH fell to 3.73 ± 0.13 (*n* = 3) after 13 days and continued to decrease to 2.97 ± 0.06 (*n* = 3) after 39 days. This likely inhibited gluconic acid production by *G. oxydans* that has an optimum growth pH 5.5–6.0 (Gupta et al., 2001). However, the solubilization of iron as a proxy for vanadium dissolution suggested promising results for metal recovery from the magnetite concentrate.

**FIGURE 5:**
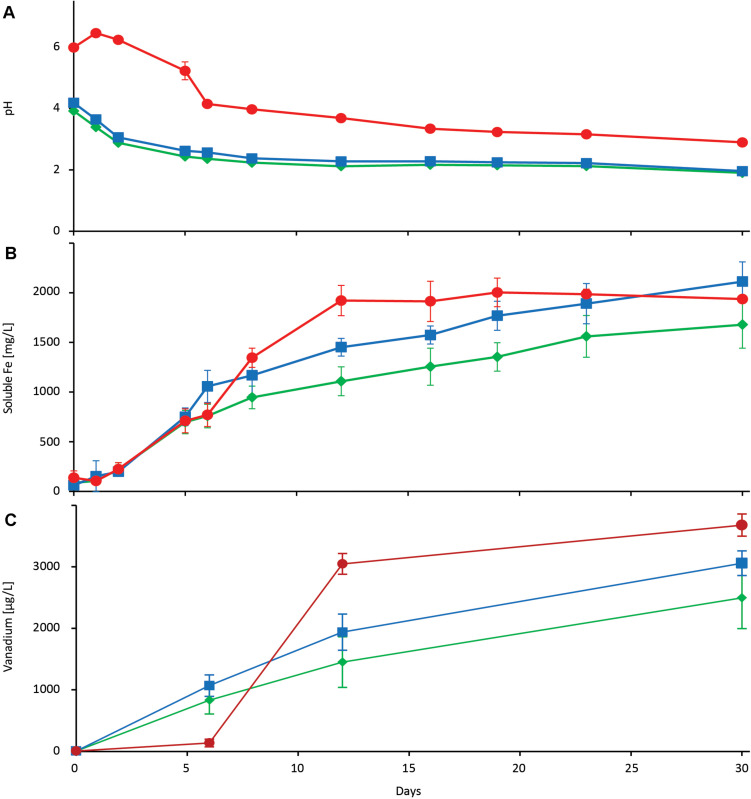
Bioleaching of 3% magnetite concentrate using *Gluconobacter oxydans* with 0.1% (wt/vol) yeast extract (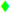), 1.0% (wt/vol) yeast extract (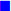), and 1.0% (wt/vol) yeast extract plus 2.0% (wt/vol) CaCO_3_ (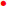). Results show pH **(A)**, soluble iron ions **(B)**, and vanadium **(C)**. Data are averages ± SD (*n* = 3).

Investigation of *G. oxydans* magnetite concentrate bioleaching with increased yeast extract (1.0% wt/vol) yielded 2112 ± 199 mg/L (9.8%) soluble iron after 30 days that was greater than with 0.1% (wt/vol) yeast extract (Students *t*-test, *p* < 0.01; Figure 5). However, once again the medium pH fell to 2.27 ± 0.01 after 12 days, which likely negatively affected the leaching rate of 128 mg/L/day (*R*^2^ = 0.95). Further amending the growth medium with 2% CaCO_3_ to pH buffer the medium yielded 1921 ± 153 mg/L soluble iron after 12 days with an iron dissolution rate of 158 mg/L/day (*R*^2^ = 0.97). However, while the pH buffering increased the leaching rate it did not increase the total dissolved iron compared to without pH buffering after 30 days (*p* = 0.11 for total dissolved iron; Figure 5). Vanadium yields after 30 days in *G. oxydans* assays with 0.1 and 1% yeast extract were 2496 ± 498 and 3059 ± 200 μg/L, respectively, confirming the difference that was observed by comparing respective iron dissolution rates. Likewise, vanadium yields were highest in medium with 2% CaCO_3_ (3678 ± 180 μg/L) indicating that glucose conversion to gluconic acid by *G. oxydans* is most efficient when the pH is efficiently buffered (Figure 5). Furthermore, vanadium dissolution in *G. oxydans* growth medium is pH dependent, since vanadium solubilization by gluconic acid occurred as soon as the pH dropped below a value of 4. Finally, the lower vanadium yields compared to iron dissolution supports that the mineral analysis in that the vanadium was present deep within the sample.

## Conclusion

Vanadium containing environments contain microorganisms that can potentially be utilized for bioleaching of magnetite. The presence of these microbes may also explain the elevated levels of vanadium in water samples at the sampling sites. Magnetite leaching using acidophiles under aerobic and anaerobic culturing did not result in significant accumulation of dissolved iron, which functions as a proxy for vanadium release. Additionally, bioleaching with *S. loihica* PV-4 did not solubilize a significant fraction of the available iron in the magnetite concentrate. However, chemical leaching with oxalic and gluconic acids showed significant iron release that led to the evaluation of *G. oxydans*-mediated biogenic gluconic acid leaching that resulted in a maximum yield of 9.8% of the available iron and 3.3% of the vanadium. Amendment of the growth medium with CaCO_3_ to buffer the pH resulted in an increased leaching rate, but did not result in additional total iron solubilization. Future work to increase the yield and efficiency of the magnetite bioleaching may be directed toward optimizing the gluconic acid production by *G. oxydans* along with other organic acid producing strains such as *A. niger*.

## Data Availability Statement

The raw data supporting the conclusions of this article will be made available by the authors, without undue reservation. 16S rRNA gene sequences are available at NCBI Sequence Read Archive (SRA) under the BioProject ID PRJNA700423.

## Author Contributions

TL and MD conceived the study. RZ, TL, IW, RE, and MD collected the samples. RZ, TL, and RE analyzed the mineral samples. ST and LS prepared and analyzed the environmental 16S rRNA gene data. SB, VS, and NW carried out the (bio)leaching experiments. SB, ST, and MD drafted the manuscript that was approved by all authors. All authors contributed to the article and approved the submitted version.

## Conflict of Interest

The authors declare that the research was conducted in the absence of any commercial or financial relationships that could be construed as a potential conflict of interest.

## Footnotes

1 www.scilifelab.se

## Supplementary Material

Supplementary File 1 Sample names and characteristics for mineralogical and microbiological analyses.

Supplementary File 2 Analysis of vanadium containing environments showing data from **(A)** water samples and **(B)** XRF results for solid samples at Bruvann and Mustavaara tailings deposits for the major (%) and minor components (ppm). The sample designations are as described in Supplementary File 1.

Supplementary File 3 Rarefaction curves showing the sequencing depth for the different samples. The sample designations are as described in Supplementary File 1.

Supplementary File 4 Number of observed species, richness estimator Chao1, and Shannon diversity index for the different samples (without rarefying). Values in brackets are underestimations because of low sequencing depth for these samples.

Supplementary File 5 Graphs of the microbial community composition based on the relative abundances of classes **(A)**, orders **(B)**, families **(C)**, and genera [**(D)**, >1%]. For the stacked bar graphs, the remaining proportion to 100% (1.0) include low-abundant taxa constituting <1% of the relative abundance.

Supplementary File 6 Chemical composition of magnetite concentrate by XRF analysis.

Supplementary File 7 XRD pattern of magnetite concentrate.

Supplementary File 8 Surface elemental composition (weight %) of magnetite concentrate by XPS.

Supplementary File 9 Fe2p **(A)** and Ti2p **(B)** high-resolution XPS spectra of magnetite concentrate.
