# Supplementary material for: Towards Bioleaching of a Vanadium Containing Magnetite for Metal Recovery
Source: Front Microbiol. 2021 Jun 30;12:693615. doi: 10.3389/fmicb.2021.693615 (PMC8278310; doi:10.3389/fmicb.2021.693615)
Supplement: Supplementary File 1 — Sample names and characteristics for mineralogical and microbiological analyses. [file Data_Sheet_1.docx]

Towards bioleaching of a vanadium containing
magnetite for metal recovery

Sören Bellenberg, Stephanie Turner, Laura Seidel, Nathan van Wyk, Ruichi Zhang,
Varvara Sachpazidou, Rodrigo F. Embile Jr., Ingar Walder, Tiina Leiviskä, & Mark Dopson

Supplementary Material

**Supplemental File S1 |** Sample names and characteristics for mineralogical and microbiological analyses.

| **Sample**  **name** | **Replicate**  **number** | **Mine** | **Coordinates** | **Sample type** | **Comments** |
| --- | --- | --- | --- | --- | --- |
| **Samples for mineralogical/chemical analysis** | | | | | |
| BRU_S1 | 1 | Bruvann closed mine, NO | 68°19’54”N 16°56’37”E | Waste rock | Solid samples collected from the Bruvann mine by combining material from the upper 15 cm of the waste dump |
| BRU_S2 | 2 | Bruvann closed mine, NO | 68°20’02”N 16°56’30”E | Waste rock |  |
| BRU_S3 | 3 | Bruvann closed mine, NO | 68°20’02”N 16°49’29”E | Waste rock |  |
| BRU_S4 | 4 | Bruvann closed mine, NO | 68°19’54”N 16°56’37”E | Waste rock |  |
| BRU_S5 | 5 | Bruvann closed mine, NO | 68°20’02”N 16°56’30”E | Waste rock |  |
| BRU_S6 | 6 | Bruvann closed mine, NO | 68°20’02”N 16°49’29”E | Waste rock |  |
| BRU_L1 | 1 | Bruvann closed mine, NO | 68°19’54”N 16°56’37”E | Water | Water sample from stream emanating from the closed mine entrance |
| BRU_L2 | 2 | Bruvann closed mine, NO | 68°20’02”N 16°56’30”E | Water |  |
| BRU_L3 | 1 | Bruvann tailings deposit, NO | 68°20’02”N 16°49’29”E | Water | Water sample from stream leading from the mine tailings deposit |
| BRU_L4 | 1 | Bruvann, NO | 68°20’02”N 16°49’29”E | Water | Water from a laboratory leaching test |
| MV_S1 | 1 | Mustavaara, FI | 65.8° N, 28.1° E | Solid waste | Mustavaara tailings site |
| MV_S2 | 2 | Mustavaara, FI | 65.8° N, 28.1° E | Solid waste |  |
| MV_L1 | 1 | Mustavaara, FI | 65.8° N, 28.1° E | Water | Settling basin in Mustavaara tailings site |
| MV_L2 | 2 | Mustavaara, FI | 65.8° N, 28.1° E | Water |  |
| Ti_L1 | 1 | Titania, NO | 58°2’ N, 06°2’ E | Water | Titania mine water |
|  |  |  |  |  |  |
|  |  |  |  |  |  |
| **Samples for microbiological analysis** | | | | | |
| BRU_S7 | 1 | Bruvann, NO | 68°20’02”N 16°56’30”E | Sediment | Sediment sample from stream emanating from the closed mine entrance |
| BRU_S8 | 2 | Bruvann, NO | 68°20’02”N 16°49’29”E | Sediment |  |
| BRU_L1 | 1 | Bruvann closed mine, NO | 68°19’54”N 16°56’37”E | Water (filter) | Water sample from stream emanating from the closed mine entrance |
| BRU_L2 | 2 | Bruvann closed mine, NO | 68°20’02”N 16°56’30”E | Water (filter) |  |
| BRU_L3 | 1 | Bruvann tailings deposit, NO | 68°20’02”N 16°49’29”E | Water (filter) | Water sample from stream leading from the mine tailings deposit |
| BRU_L4 | 1 | Bruvann, NO | 68°20’02”N 16°49’29”E | Water (filter) | Water from a laboratory leaching test |
| MV_S3 | 1 | Mustavaara, FI | 65.8° N, 28.1° E | Solid waste | Mustavaara tailing site |
| MV_S4 | 2 | Mustavaara, FI | 65.8° N, 28.1° E | Solid waste |  |
| MV_S5 | 3 | Mustavaara, FI | 65.8° N, 28.1° E | Solid waste |  |
| MV_S6 | 4 | Mustavaara, FI | 65.8° N, 28.1° E | Solid waste |  |
| MV_S7 | 5 | Mustavaara, FI | 65.8° N, 28.1° E | Solid waste |  |
| MV_S8 | 6 | Mustavaara, FI | 65.8° N, 28.1° E | Solid waste |  |
| MV_L3 | 1 | Mustavaara, FI | 65.8° N, 28.1° E | Water (filter) | Settling basin in Mustavaara tailings site |
| MV_L4 | 2 | Mustavaara, FI | 65.8° N, 28.1° E | Water (filter) |  |
| MV_L5 | 3 | Mustavaara, FI | 65.8° N, 28.1° E | Water (filter) |  |
| Ti_L1 | 1 | Titania, NO | 58°2’ N, 06°2’ E | Water (filter) | Titania mine water |

**Supplemental File S2 |** Analysis of vanadium containing environments showing data from (A) water samples and (B) XRF results for solid samples at Bruvann and Mustavaara tailings deposits for the major (%) and minor components (ppm). The sample designations are as described in Supplemental File S1.

**A**

| **Sample name** | **Replicate** | **pH** | **Conductivity**  **(µS/cm)** | **Vanadium**  **(µg/L)** |
| --- | --- | --- | --- | --- |
| BRU_L1 | 1 | 7.43 | 266 | No data |
| BRU_L2 | 2 | 7.21 | 420 | No data |
| BRU_L3 | 1 | 6.75 | 610 | No data |
| BRU_L4 | 1 | 5.8 | 28.3 | No data |
| MV_L1 | 1 | 6.42 | 202 | 9.42 |
| MV_L2 | 2 | 6.54 | 260 | 9.74 |
| Ti_L1 | 1 | 1.69 | 13540 | 526 |

**B**

|  | BRU_S1 | BRU_S2 | BRU_S3 | BRU_S4 | BRU_S5 | BRU_S6 | MV_S1 | MV_S2 |
| --- | --- | --- | --- | --- | --- | --- | --- | --- |
| F (%) | 0.52 | 0.58 | 0.44 | 0.67 | 0.67 | 0.00 | 0.08 | 0.05 |
| Na_2_O (%) | 0.52 | 0.77 | 0.21 | 1.04 | 0.60 | 0.21 | 2.26 | 2.37 |
| MgO (%) | 21.83 | 21.22 | 27.52 | 16.26 | 22.74 | 32.02 | 6.41 | 6.12 |
| Al_2_O3 (%) | 6.03 | 7.81 | 3.41 | 11.28 | 7.13 | 3.08 | 14.21 | 14.80 |
| SiO_2_ (%) | 37.28 | 42.69 | 35.31 | 43.40 | 40.71 | 37.16 | 46.01 | 47.07 |
| P_2_O_5_ (%) | 0.04 | 0.06 | 0.01 | 0.07 | 0.03 | 0.00 | 0.03 | 0.03 |
| S (%) | 0.76 | 0.24 | 0.31 | 0.08 | 0.37 | 0.06 | 0.13 | 0.05 |
| Cl (ppm) | 0 | 0 | 0 | 0 | 0 | 0 | 367 | 362 |
| K_2_O (%) | 0.38 | 0.51 | 0.13 | 0.46 | 0.29 | 0.08 | 0.28 | 0.30 |
| CaO (%) | 2.56 | 3.66 | 1.37 | 6.16 | 3.74 | 1.40 | 10.92 | 11.19 |
| Sc (ppm) | 11 | 16 | 8 | 15 | 12 | 6 | 57 | 59 |
| TiO_2_ (%) | 0.27 | 0.39 | 0.20 | 0.51 | 0.29 | 0.14 | 2.28 | 1.48 |
| V (ppm) | 122 | 146 | 59 | 155 | 161 | 79 | 1029 | 755 |
| Cr_2_O_3_ (%) | 0.16 | 0.18 | 0.17 | 0.19 | 0.20 | 0.06 | 0.00 | 0.00 |
| MnO (%) | 0.15 | 0.16 | 0.17 | 0.16 | 0.16 | 0.19 | 0.19 | 0.16 |
| FeO (%) | 16.47 | 12.10 | 16.28 | 11.01 | 12.93 | 15.68 | 15.03 | 12.59 |
| Ni (ppm) | 1979 | 1341 | 3699 | 553 | 1377 | 766 | 128 | 125 |
| Cu (ppm) | 1096 | 292 | 686 | 114 | 420 | 52 | 701 | 869 |
| Zn (ppm) | 106 | 90 | 100 | 86 | 93 | 108 | 128 | 134 |
| Ga (ppm) | 7 | 9 | 4 | 11 | 7 | 4 | 23 | 23 |
| As (ppm) | 153 | 98 | 260 | 46 | 116 | 32 | 0 | 0 |
| Rb (ppm) | 25 | 26 | 17 | 20 | 18 | 9 | 2 | 3 |
| Sr (ppm) | 60 | 96 | 8 | 159 | 80 | 4 | 273 | 302 |
| Y (ppm) | 7 | 11 | 4 | 12 | 8 | 4 | 12 | 13 |
| Zr (ppm) | 35 | 51 | 19 | 44 | 30 | 14 | 25 | 21 |
| Mo (ppm) | 5 | 1 | 5 | 1 | 3 | 5 | 0 | 0 |
| Sn (ppm) | 6 | 6 | 6 | 6 | 6 | 6 | 0 | 0 |
| Sb (ppm) | 42 | 42 | 42 | 42 | 42 | 42 | 39 | 39 |
| Cs (ppm) | 11 | 6 | 5 | 2 | 0 | 2 | 13 | 14 |
| Ba (ppm) | 119 | 103 | 50 | 86 | 78 | 29 | 108 | 132 |
| La (ppm) | 16 | 16 | 16 | 16 | 16 | 16 | 13 | 13 |
| Ce (ppm) | 36 | 28 | 22 | 29 | 13 | 15 | 13 | 24 |
| Pr (ppm) | 5 | 5 | 5 | 5 | 5 | 5 | 6 | 6 |
| Nd (ppm) | 21 | 22 | 17 | 17 | 11 | 12 | 12 | 16 |
| Gd (ppm) | 216 | 138 | 223 | 119 | 151 | 192 | 0 | 0 |
| Hf (ppm) | 2 | 4 | 2 | 3 | 2 | 1 | 2 | 2 |
| Ta (ppm) | 1 | 7 | 4 | 4 | 5 | 1 | 0 | 4 |
| Pb (ppm) | 26 | 15 | 27 | 10 | 15 | 12 | 8 | 6 |
| Bi (ppm) | 18 | 12 | 20 | 11 | 14 | 12 | 4 | 5 |
| Th (ppm) | 2 | 2 | 2 | 2 | 2 | 2 | 2 | 2 |
| U (ppm) | 2 | 2 | 2 | 3 | 2 | 2 | 3 | 4 |

**Supplemental File S3 |** Rarefaction curves showing the sequencing depth for the different samples. The sample designations are as described in Supplemental File S1.

**
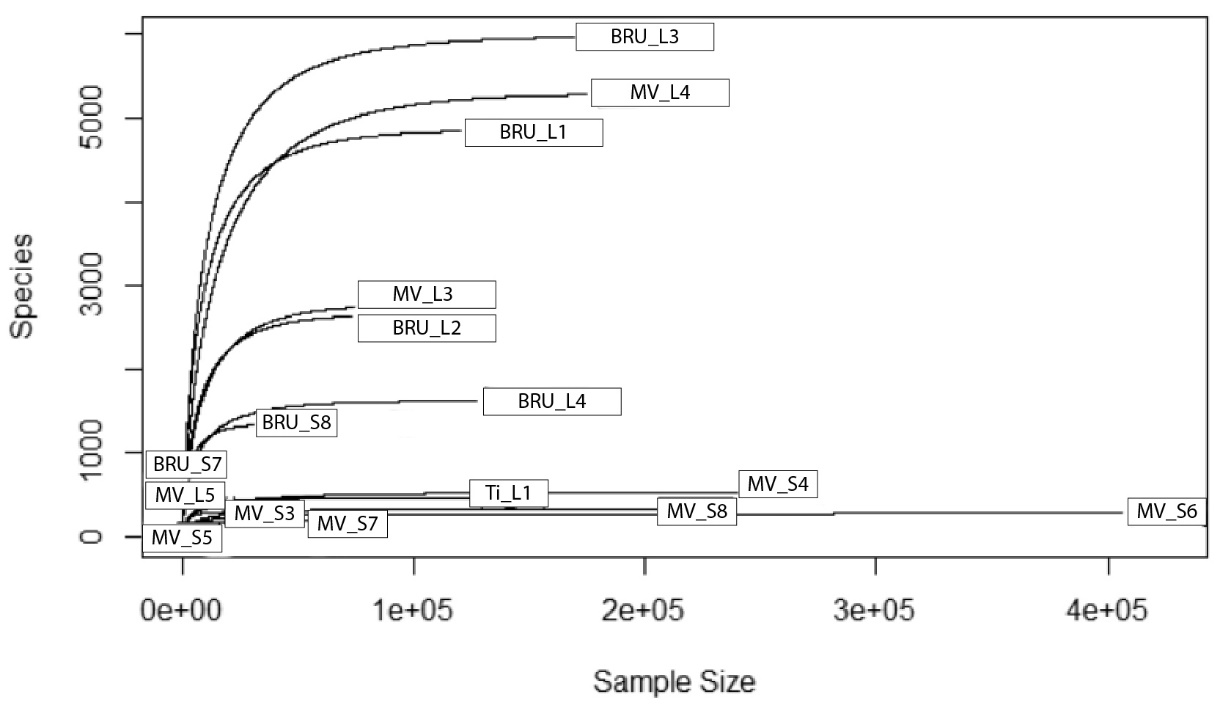
**

**Supplemental File S4 |** Number of observed species, richness estimator Chao1, and Shannon diversity index for the different samples (without rarefying). Values in brackets are underestimations because of low sequencing depth for these samples.

| **Sample name** | **Observed** | **Chao1** | **Shannon** |
| --- | --- | --- | --- |
| BRU_L1 | 4852 | 4854 | 7.69 |
| BRU_L2 | 2646 | 2647 | 6.38 |
| BRU_L3 | 5972 | 5977 | 7.85 |
| BRU_L4 | 1617 | 1617 | 5.66 |
| BRU_S7 | 1356 | 1356 | 6.23 |
| BRU_S8 | 695 | 716 | 5.40 |
| MV_L3 | 2758 | 2760 | 6.04 |
| MV_L4 | 5282 | 5285 | 6.87 |
| MV_L5 | (493) | (493) | (5.37) |
| MV_S3 | 296 | 297 | 3.81 |
| MV_S4 | 525 | 526 | 3.89 |
| MV_S5 | (30) | (30) | (2.92) |
| MV_S6 | 271 | 271 | 3.29 |
| MV_S7 | 185 | 185 | 3.18 |
| MV_S8 | 320 | 320 | 3.33 |
| Ti_L1 | 462 | 462 | 3.56 |

**Supplemental File S5 |** Graphs of the microbial community composition based on the relative abundances of classes (A), orders (B), families (C), and genera (D; > 1%). For the stacked bar graphs, the remaining proportion to 100% (1.0) include low-abundant taxa constituting < 1% of the relative abundance.

**A**

**
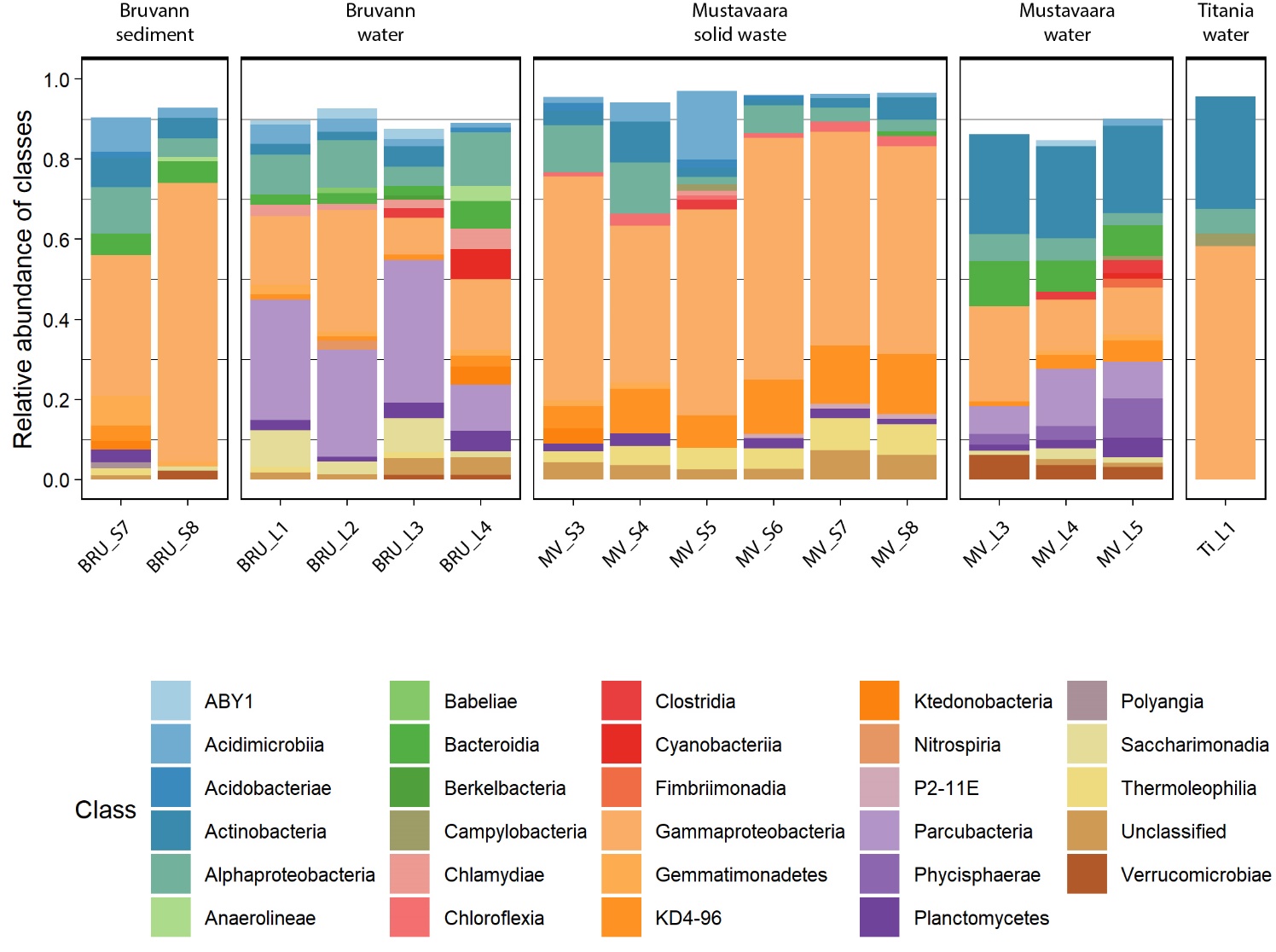
**

**B**

**
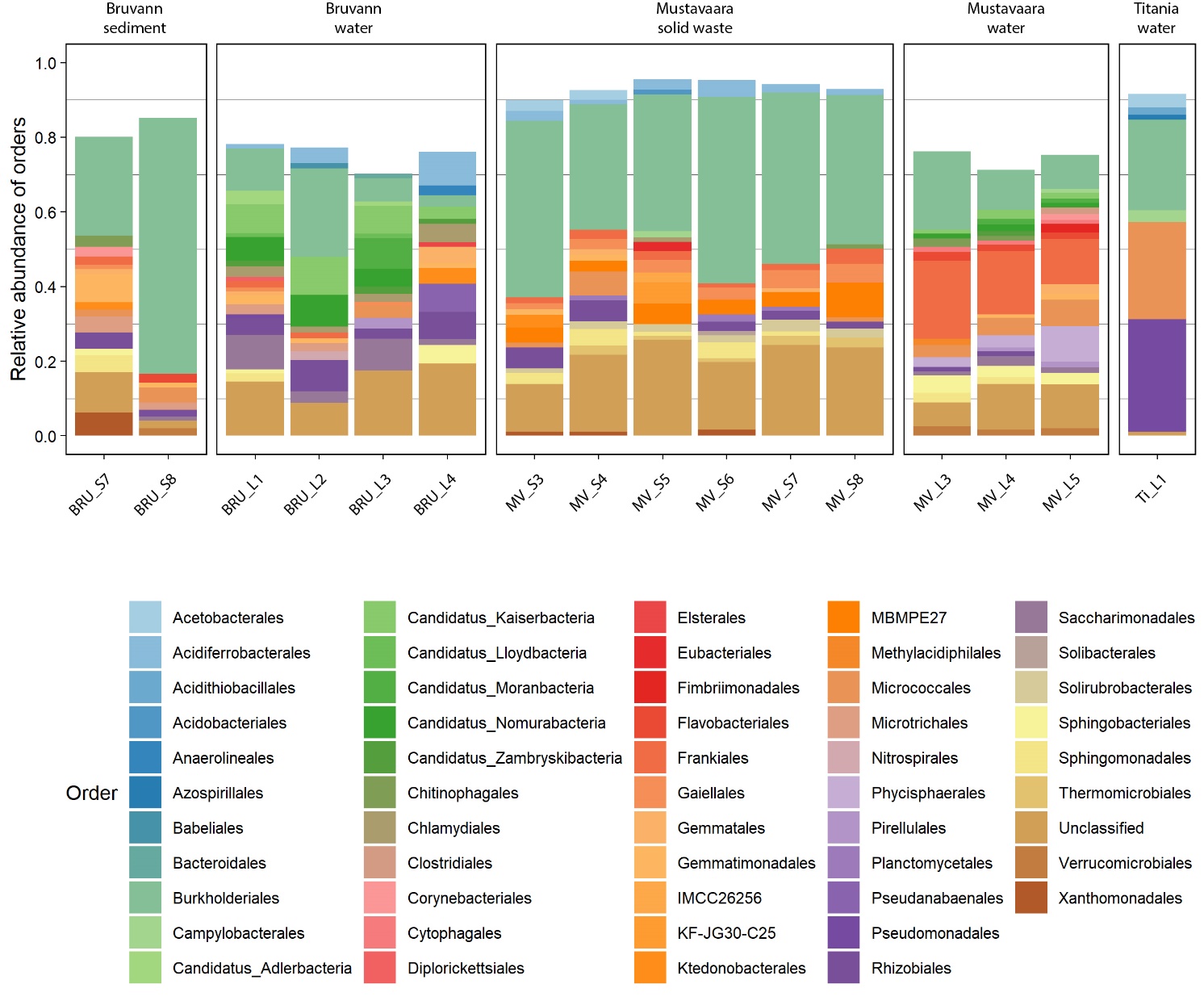
**

**C**

**
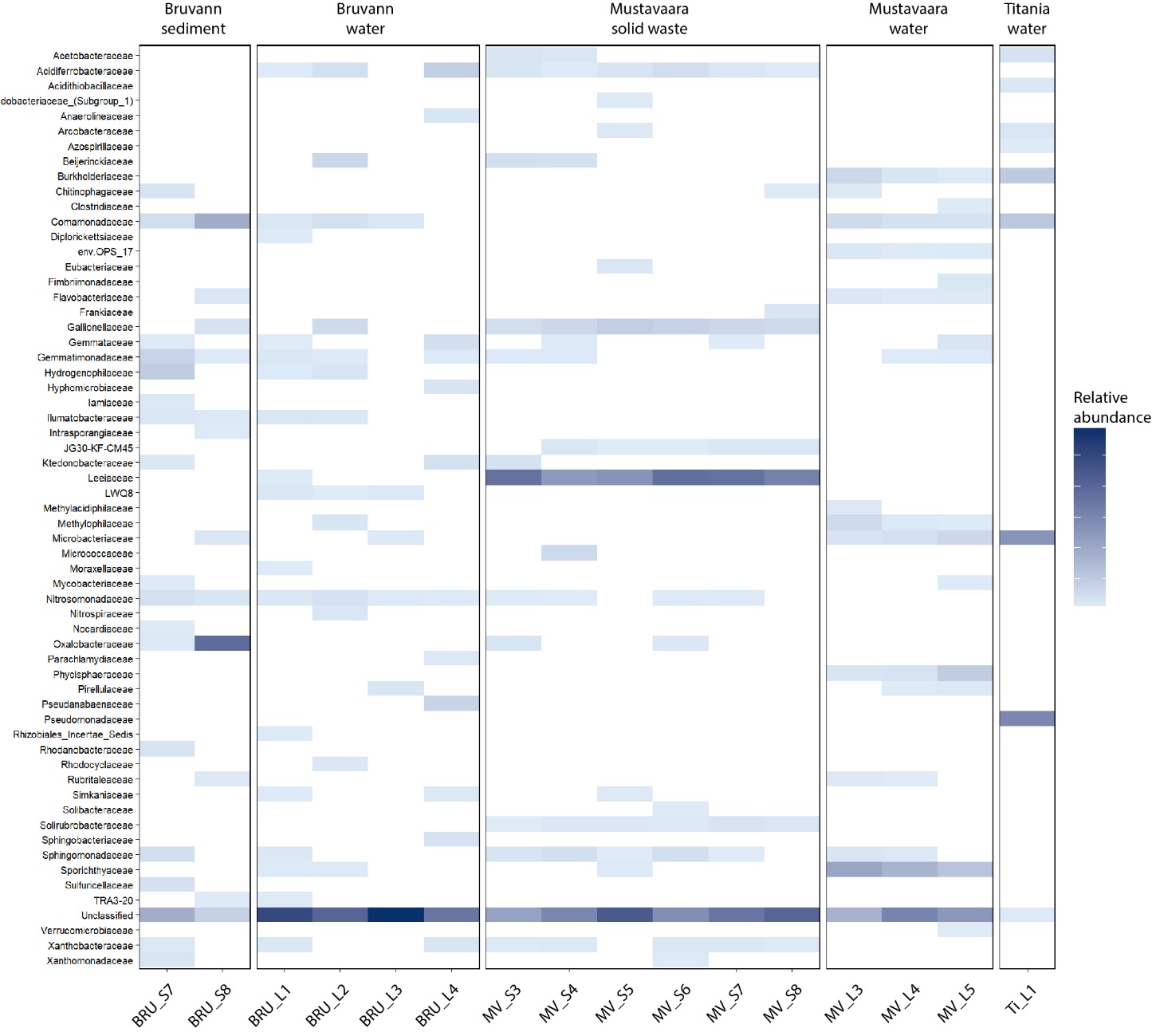
**

**D**

**
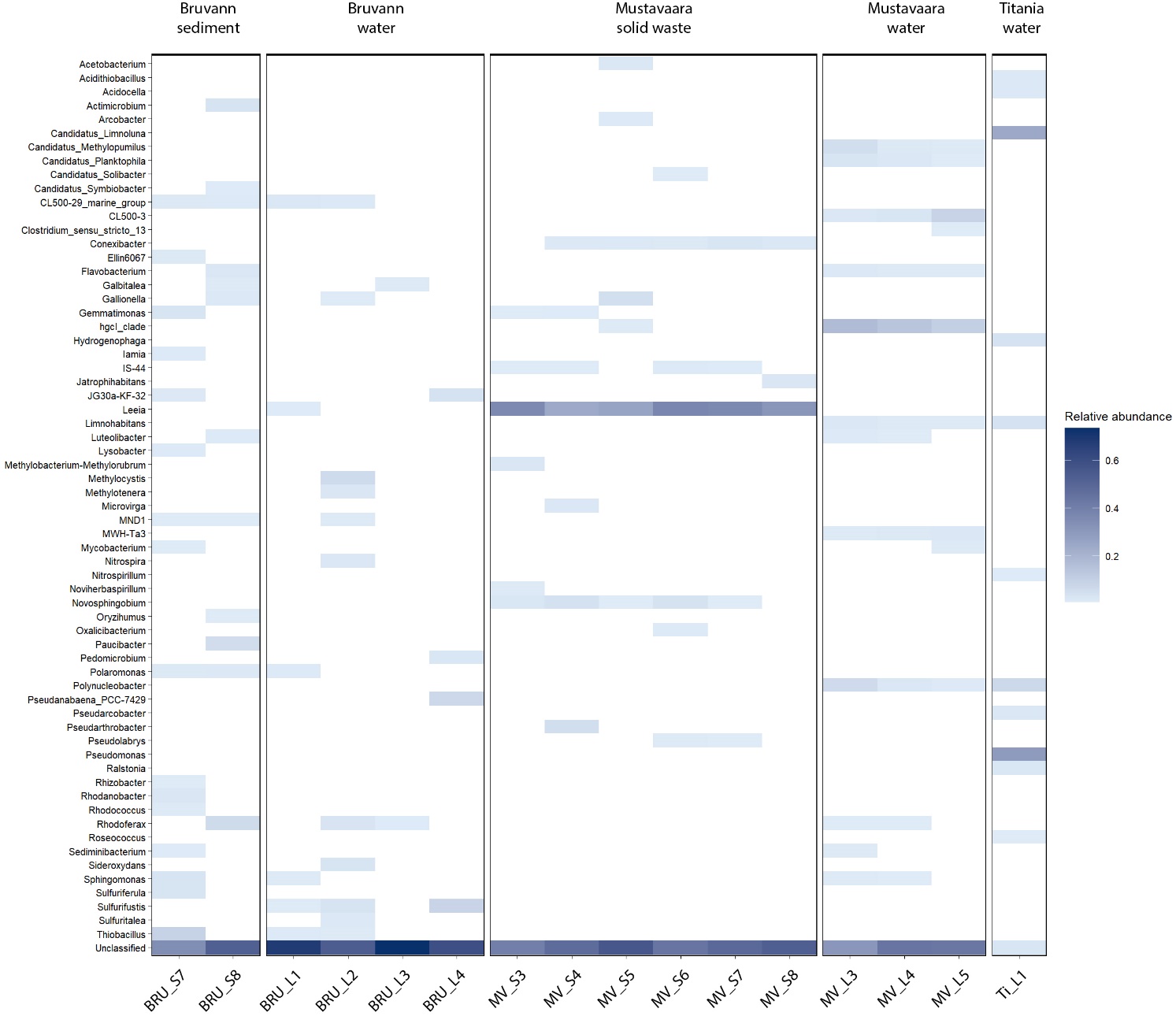
**

**Supplemental File S6 |** Chemical composition of magnetite concentrate by XRF analysis.

| **Component** | **%** | **Component** | **ppm** | **Component** | **ppm** |
| --- | --- | --- | --- | --- | --- |
| FeO | 82.53 | V | 3739 | La | 15 |
| SiO_2_ | 6.15 | Zn | 1334 | Pb | 9 |
| MgO | 4.63 | Ni | 742 | Bi | 7 |
| TiO_2_ | 4.55 | Zr | 135 | Y | 6 |
| Al_2_O_3_ | 3.38 | Cu | 117 | Sn | 6 |
| Cr_2_O_3_ | 1.02 | Ga | 82 | Sc | 6 |
| CaO | 0.46 | Ce | 39 | Sr | 5 |
| S | 0.28 | Cs | 38 | Mo | 5 |
| Na_2_O | 0.24 | Sb | 33 | Pr | 5 |
| K_2_O | 0.05 | Nd | 25 | Th | 2 |
| MnO | 0.04 | As | 22 | U | 1 |

**Supplemental File S7 |** XRD pattern of magnetite concentrate.

**

**

**Supplemental File S8 |** Surface elemental composition (weight %) of magnetite concentrate by XPS.

| **Component** | **%** |
| --- | --- |
| O1s | 47.0 |
| Fe2p | 23.2 |
| Si2p | 10.3 |
| Mg1s | 8.9 |
| S2p | 3.9 |
| C1s | 3.4 |
| Al2p | 2.0 |
| Na1s | 0.9 |
| Ti2p | 0.5 |

**Supplemental File S9 |** Fe2p (A) and Ti2p (B) high-resolution XPS spectra of magnetite concentrate.

**

**
